# Resection margins in transoral oropharyngeal surgery: comparative analysis of robotic and VITOM Eagle–assisted exoscopic approaches

**DOI:** 10.3389/fonc.2026.1919981

**Published:** 2026-09-16

**Authors:** Erika Crosetti, Andrea Lorenzi, Giulia Arrigoni, Ilaria Bertotto, Adolfo Suriani, Nara Stella, Giovanni Succo

**Affiliations:** 1 ENT Department–Head and Neck Cancer Unit, San Giovanni Bosco Hospital, Turin, Italy; 2 Department of Oncology, University of Turin, Turin, Italy; 3 Department of Surgical Sciences, University of Turin, Turin, Italy; 4 Department of Mechanical and Aerospace Engineering, Politecnico di Torino, Turin, Italy; 5 Radiology Service, INOC – Istituto Nazionale Oncologico Candiolo, Candiolo, Italy; 6 Pathology Service, San Giovanni Bosco Hospital, Turin, Italy; 7 Department of Clinical and Biological Sciences, University of Turin, Orbassano, Italy

**Keywords:** head and neck oncology, margin adequacy, oropharyngeal squamous cell carcinoma, surgical margins, three-dimensional exoscopy, transoral robotic surgery, transoral surgery, VITOM Eagle

## Abstract

**Background:**

Transoral robotic surgery (TORS) is widely regarded as the current benchmark minimally invasive approach for early-stage oropharyngeal squamous cell carcinoma (OPSCC), although access remains limited by cost and infrastructure. Three-dimensional exoscopic surgery (3Des) has recently emerged as a lower-cost, team-shared alternative, but clinical evidence in OPSCC is still limited. This study compared the pathological radicality and surgical margin adequacy of transoral resections performed using a robotic platform versus a 3D exoscope.

**Methods:**

This retrospective cohort study included 94 consecutive patients with OPSCC treated between 2016 and 2026 by transoral surgery using either the da Vinci Xi system (robotic group, R; n = 75) or the VITOM Eagle 3D exoscope (exoscopic group, E; n = 19). Margins were classified as positive (tumor at inked margin), close (<1 mm), or negative (≥1 mm). Superficial and deep margin distributions, perioperative outcomes, and early oncologic events were compared between platforms.

**Results:**

Positive margins occurred in 14.7% of R patients and 5.3% of E patients. Close margins (<1 mm) were observed in 12.0% and 26.3%, respectively, while margins ≥1 mm were achieved in 73.3% and 68.4%. Platform-specific analysis demonstrated comparable superficial and deep margin control (superficial ≥1 mm: 85.3% R vs. 73.7% E, *p* = 0.30; deep ≥1 mm: 77.3% R vs. 73.7% E, *p* = 0.77), with no significant differences in positive margin rates between platforms (superficial, *p* = 1.00; deep, *p* = 0.68). No procedure-related conversions occurred in the E-group. At a median follow-up of 34 months (47 mo R vs. 6 mo E), no statistically significant difference in early recurrence was observed (13.3% vs. 5.3%); however, this comparison is limited by the substantially shorter follow-up in the E-group.

**Conclusions:**

Three-dimensional exoscopic transoral surgery achieved pathological margin status and perioperative safety comparable to those obtained with the robotic platform. By combining stereoscopic visualization, shared operative field view, and compatibility with CO_2_ laser resection, exoscopy represents a feasible and scalable minimally invasive platform for centers without routine robotic access. Prospective multicenter studies with longer follow-up are warranted to formally assess non-inferiority in terms of oncologic and functional outcomes.

## 1 Introduction

Oropharyngeal squamous cell carcinoma (OPSCC) represents one of the most epidemiologically relevant malignancies of the head and neck region, with its incidence steadily increasing over recent decades, particularly in Western countries. This trend is largely attributable to the increasing prevalence of human papillomavirus (HPV) infection, which has profoundly reshaped the epidemiology, clinical presentation, and prognosis of the disease (1). Alongside HPV-related tumors, however, a substantial proportion of OPSCCs remains associated with traditional risk factors such as alcohol and tobacco consumption, typically exhibiting a less favorable clinical course (2).

The management of OPSCC relies on a multimodal approach integrating surgery, radiotherapy, and chemotherapy, tailored according to tumor stage, biological features, and patient-related factors. In recent years, technological advances have promoted the increasingly widespread adoption of minimally invasive transoral surgical techniques, aiming to reduce the morbidity associated with conventional open approaches while preserving oncologic radicality (3). When feasible, these techniques may also support de-intensified and preferably single-modality or, at most, bimodality treatment strategies (surgery followed by radiotherapy) (4, 5).

Transoral robotic surgery (TORS), first introduced in 2005, has enabled safe access to anatomically complex regions through high-definition three-dimensional (3D) visualization and dedicated instrumentation. Although favorable oncologic and functional outcomes have been reported, its widespread adoption remains limited by high acquisition and maintenance costs, as well as by the need for dedicated infrastructure and trained personnel (6).

In parallel, new technological tools for transoral approaches have been introduced, including the exoscope—a digital optical system that provides magnified 3D visualization of the operative field via an external camera projecting onto high-definition monitors. This technology offers promising advantages in terms of versatility, ergonomics, and intraoperative image sharing (7, 8). Among the currently available systems, the VITOM Eagle represents one of the most recent innovations, distinguished by excellent 3D image quality and magnification, optimal ergonomics, enhanced opportunities for educational image sharing, and lower costs compared with robotic platforms (9, 10). Nevertheless, clinical experience specifically concerning its application in oropharyngeal surgery remains limited.

Within oropharyngeal oncologic surgery, one of the most debated aspects concerns surgical margins, which are a key prognostic factor for local recurrence and overall survival (11, 12). Achieving adequate margins is crucial to minimize the risk of residual or recurrent disease and to guide indications for postoperative adjuvant therapy. The primary objective of oncologic surgery is to obtain radical resection with histologically negative margins, the required clearance depending on tumor site and biological behavior. This goal generally allows avoidance of trimodal treatment (surgery followed by chemoradiotherapy), which is often required in the presence of positive margins. However, unlike other head and neck subsites, there is no universal consensus regarding optimal margin thresholds and anatomical adequacy criteria in OPSCC (13). Their assessment may also be influenced by multiple technical and pathological factors, including cutting device characteristics, tissue shrinkage phenomena, and intraoperative specimen handling (14, 15).

Given the relatively recent introduction and limited adoption of three-dimensional exoscopic surgery (3Des), it is particularly important, from both ethical and oncologic perspectives, to compare the two minimally invasive transoral approaches. Specifically, it is necessary to determine whether resections performed using 3Des achieve pathological radicality and surgical margin adequacy comparable to those obtained with robotic surgery, which is currently regarded as the reference standard.

The present study aimed to compare pathological margin status, perioperative outcomes, and early oncologic events in a cohort of patients with OPSCC undergoing minimally invasive transoral surgery using either the da Vinci Xi robotic platform or the VITOM Eagle 3D exoscopic system.

## 2 Materials and methods

A retrospective observational cohort study was conducted including patients with OPSCC treated by transoral surgical resection using either the da Vinci Xi robotic platform or the VITOM Eagle 3D exoscopic system. The primary objective was to compare resection margin status between robotic and exoscopic transoral approaches and to evaluate their feasibility and safety in routine clinical practice.

### 2.1 Study population

A total of 94 consecutive patients with OPSCC were included and treated between 2016 and 2026. All patients underwent a standardized preoperative assessment within the three weeks preceding surgery, including clinical examination, nutritional status evaluation, videoendoscopy (white light and narrow band imaging [NBI]), head and neck CT and/or MRI, and histopathological confirmation by biopsy.

All cases were prospectively discussed at the institutional multidisciplinary head and neck tumor board. The indication for transoral surgery was established by multidisciplinary consensus based on tumor extent, radiological findings, anticipated functional outcomes, patient comorbidities, and, where appropriate, patient preference. Once the decision to proceed with surgery had been made, patients underwent either TORS or transoral resection using 3Des, depending on platform availability and the progressive implementation of the exoscopic system during the study period. No platform-specific selection criteria based on tumor characteristics were applied.

Patients were stratified according to the surgical platform used. Seventy-five patients (79.8%) underwent transoral surgery using the da Vinci Xi robotic system (Intuitive Surgical, Sunnyvale, CA, USA) and constituted the robotic group (R-group), whereas 19 patients (20.2%) were treated using the VITOM Eagle 3D exoscopic system (Karl Storz, Tuttlingen, Germany) and constituted the exoscopic group (E-group).

All procedures were performed in accordance with standard oncologic and functional principles, the ethical standards of the relevant institutional and national research committees, and the Declaration of Helsinki and its subsequent amendments. Under applicable national and institutional regulations, formal ethics committee approval was not required for this retrospective study. All patients provided written informed consent for the use of their anonymized clinical data for scientific purposes.

Procedures in both groups were performed by the same dedicated head and neck surgical team, whose members had extensive prior experience in transoral oncologic surgery. The introduction of the VITOM Eagle system represented the adoption of a new visualization platform rather than a new surgical technique. Accordingly, patient selection, oncologic resection principles, specimen orientation, and pathological assessment remained standardized throughout the study period, thereby limiting operator-related variability.

In the E-group, procedures were performed using the VITOM Eagle 3D exoscopic system. This external optical device was positioned above the surgical field and enclosed within a sterile disposable sheath, allowing placement in close proximity to the operative area while maintaining sterility. The system provides 4K 3D imaging through a four-sensor camera platform integrated with the IMAGE1 S system, enabling stereoscopic visualization of anatomical structures and seamless integration with endoscopic imaging. The system is equipped with enhanced zoom capabilities, allowing magnification of fine anatomical details up to 45.5×, supporting precise identification of tumor boundaries and critical structures.

The video signal was displayed on a 55-inch 3D monitor (3,840 × 2,160 pixel resolution; 16:9 aspect ratio), positioned directly in front of the primary surgeon along the operative axis and viewed by all operating room personnel using passive-polarized 3D glasses. Optical magnification was achieved using 6× optical zoom combined with 2× digital zoom, further extended by the system’s advanced zoom functions.

Camera orientation, focus, and zoom were controlled through the IMAGE1 PILOT system, foot pedals, or a 3D joystick with programmable functions, usable in both sterile and non-sterile configurations. The illumination source was aligned with the optical axis, ensuring homogeneous lighting and minimal shadowing. The typical working distance ranged from 20 to 50 cm, facilitating its use in transoral microsurgical procedures.

The exoscopic platform can be combined with a CO_2_ laser. In the E-group, superficial tumor margin delineation was performed using a CO_2_ laser (DEKA, Calenzano, Italy), allowing accurate and controlled mucosal and submucosal incision with minimal thermal damage (**Figure 1**).

**Figure 1:**
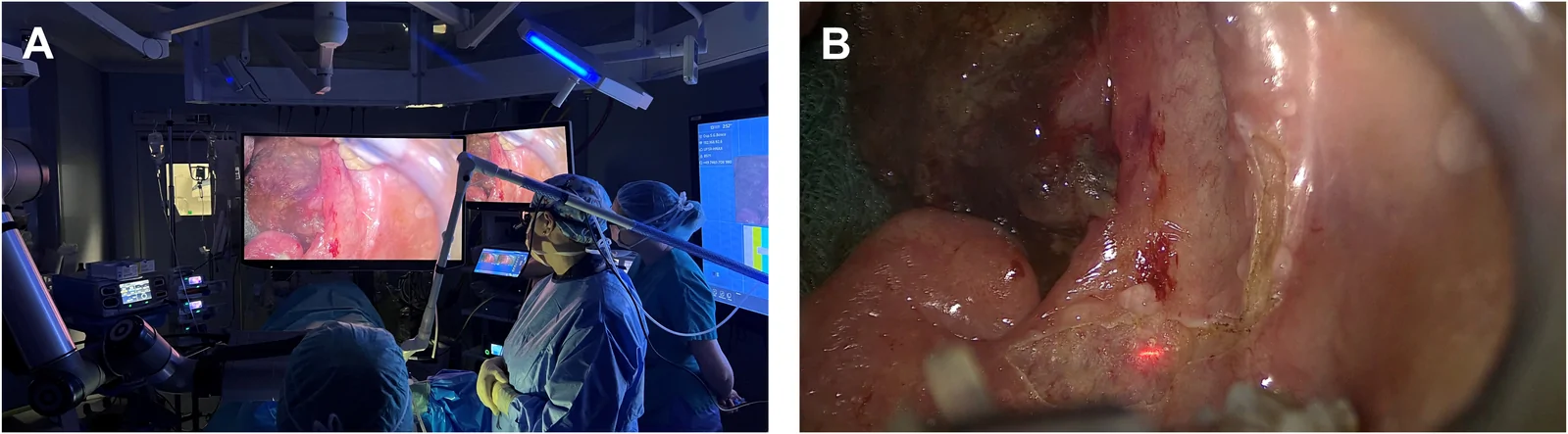
Exoscopic transoral surgery. **(A)** Intraoperative setup. On the left, the VITOM Eagle system mounted on an ARTip Cruise articulated arm and coupled with a CO_2_ laser micromanipulator, with a 55-inch 3D main monitor positioned at the foot of the operating table. On the right, the CollaboratOR platform for visualization of patient imaging and clinical data. **(B)** Surgical resection using CO_2_ laser.

In both the robotic and exoscopic groups, intraoperative frozen-section assessment was not routinely performed. Immediately after resection, all specimens were oriented by the surgical team, and the resection margins were systematically inked according to a predefined color-coded scheme. The specimens were then fixed in Bouin’s solution (picric acid–formaldehyde–acetic acid fixative) and submitted for pathological evaluation, with direct communication between the surgical and pathology teams to ensure correct specimen orientation and margin identification (**Figure 2**).

**Figure 2:**
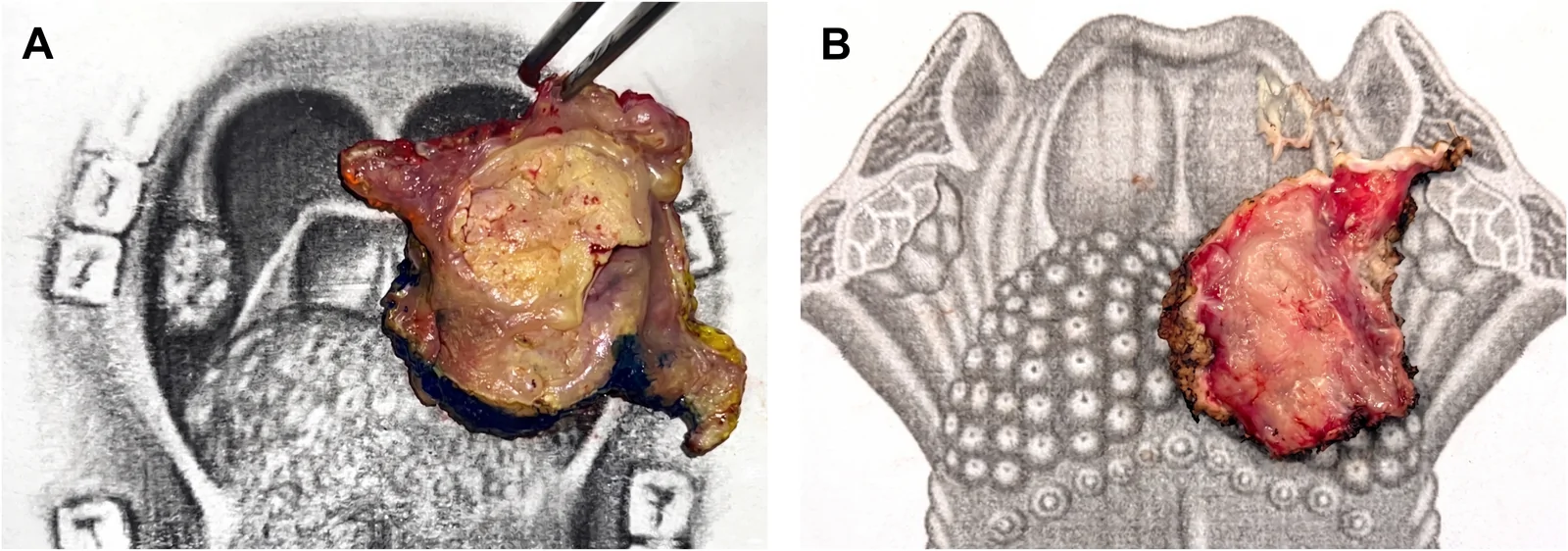
**(A)** Oropharyngectomy specimen. **(B)** Base of tongue resection specimen.

### 2.2 Statistical analysis

Continuous variables were summarized as mean ± standard deviation (SD), median, interquartile range (IQR; 25th–75th percentile), and minimum–maximum range, as appropriate. Categorical variables were reported as absolute frequencies and percentages. Between-group comparisons for continuous variables were performed using Student’s *t*-test for independent samples or Welch’s *t*-test when the assumption of equal variances was not met. The Mann–Whitney *U* test was used for non-normally distributed variables. Categorical variables were compared using Pearson’s χ² test or Fisher’s exact test, as appropriate.

To identify independent predictors of positive surgical margins, a multivariable logistic regression model using Firth’s penalized likelihood estimation was performed to minimize small-sample and sparse-data bias. Firth’s penalized logistic regression was preferred over conventional logistic regression because of the relatively low number of positive-margin events and the imbalance between the robotic and exoscopic groups, conditions under which maximum likelihood estimation may produce biased or unstable estimates. Positive surgical margin status was considered the dependent variable, whereas negative and close margins were combined as the reference category. Based on clinical relevance and to avoid model overfitting, the model included the surgical approach (3Des vs. TORS), type of transoral resection (BOT resection vs. oropharyngectomy), and pathological T category (pTis–T2 vs. pT3–T4) as independent variables. Results are reported as adjusted odds ratios (aORs) with corresponding 95% confidence intervals (95% CIs). The Firth logistic regression model was fitted using the *logistf* package (version 1.26.1) in R.

All statistical tests were two-sided, and a *p* value <0.05 was considered statistically significant. Statistical analyses were performed using R (version 4.6.1) through RStudio (version 2026.07.0; Posit Software, PBC, Boston, MA, USA; available at https://posit.co/).

## 3 Results

The demographic characteristics of the study population are reported in **Table 1**.

**Table 1:** Patient characteristics and distribution by group.

Characteristic	Category	Total (n = 94)	R-group (n = 75)	E-group (n = 19)	*p*-value
Age (y)	Mean ± SD	66.5 ± 10.1	67.5 ± 10.1	62.6 ± 9.3	0.051
	Median (IQR)	66 (59–74)	66 (59–75)	63 (57–70)	
Sex	Male	69 (73.4%)	55 (73.3%)	14 (73.7%)	1.000
	Female	25 (26.6%)	20 (26.7%)	5 (26.3%)	
CCI	Median (IQR)	3 (2–4)	3 (2–4)	3 (2–3)	0.629
Pretreated	No	81 (86.2%)	63 (84.0%)	18 (94.7%)	0.840
	RT	3 (3.2%)	3 (4.0%)	0 (0%)	
	CCRT	10 (10.6%)	9 (12.0%)	1 (5.3%)	
HPV/p16 status	Positive	64 (68.1%)	54 (72.0%)	10 (52.6%)	0.167
	Negative	30 (31.9%)	21 (28.0%)	9 (47.4%)	
Subsite of the primary tumor	Tonsil	39 (41.5%)	32 (42.7%)	7 (36.8%)	0.747
	BOT	20 (21.3%)	16 (21.3%)	4 (21.1%)	
	CUP	13 (13.8%)	11 (14.7%)	2 (10.5%)	
	GTS	10 (10.6%)	6 (8.0%)	4 (21.1%)	
	Velum	7 (7.4%)	6 (8.0%)	1 (5.3%)	
	PPW	5 (5.3%)	4 (5.3%)	1 (5.3%)	
cT	cT0	13 (13.8%)	11 (14.7%)	2 (10.5%)	0.323
	cTis	1 (1.1%)	1 (1.3%)	0 (0%)	
	cT1	40 (42.6%)	31 (41.3%)	9 (47.4%)	
	cT2	31 (33.0%)	27 (36.0%)	4 (21.1%)	
	cT3	9 (9.6%)	5 (6.7%)	4 (21.1%)	
	cT4	0 (0%)	0 (0%)	0 (0%)	
cN	cN0	32 (34.0%)	28 (37.3%)	4 (21.1%)	0.506
	cN1	45 (47.9%)	34 (45.3%)	11 (57.9%)	
	cN2	13 (13.8%)	10 (13.3%)	3 (15.8%)	
	cN3	4 (4.3%)	3 (4.0%)	1 (5.3%)	
Clinical stage	0	1 (1.1%)	1 (1.3%)	0 (0%)	0.054
	I	59 (62.8%)	48 (64.0%)	11 (57.9%)	
	II	18 (19.1%)	17 (22.7%)	1 (5.3%)	
	III	7 (7.4%)	4 (5.3%)	3 (15.8%)	
	IV	9 (9.6%)	5 (6.7%)	4 (21.1%)	
Surgical treatment	Oropharyngectomy	74 (78.7%)	60 (80.0%)	14 (73.7%)	0.542
	BOT resection	20 (21.3%)	15 (20.0%)	5 (26.3%)	
Neck dissection	No	12 (12.8%)	11 (14.7%)	1 (5.3%)	0.562
	SND	66 (70.2%)	52 (69.3%)	14 (73.7%)	
	mRND	16 (17.0%)	12 (16.0%)	4 (21.1%)	
LVI	Absent	50 (53.2%)	43 (57.3%)	7 (36.8%)	0.129
	Present	44 (46.8%)	32 (42.7%)	12 (63.2%)	
PNI	Absent	56 (59.6%)	43 (57.3%)	13 (68.4%)	0.441
	Present	38 (40.4%)	32 (42.7%)	6 (31.6%)	
ENE (pN assessed)	Absent	56 (68.3%)	43 (67.2%)	13 (72.2%)	0.780
	Present	26 (31.7%)	21 (32.8%)	5 (27.8%)	
Grading (p16^−^ only)	G1	3 (10.0%)	2 (9.5%)	1 (11.1%)	0.070
	G2	12 (40.0%)	11 (52.4%)	1 (11.1%)	
	G3	15 (50.0%)	8 (38.1%)	7 (77.8%)	
pT	pTis	4 (4.3%)	3 (4.0%)	1 (5.3%)	0.267
	pT1	41 (43.6%)	34 (45.3%)	7 (36.8%)	
	pT2	40 (42.6%)	33 (44.0%)	7 (36.8%)	
	pT3	6 (6.4%)	3 (4.0%)	3 (15.8%)	
	pT4	3 (3.2%)	2 (2.7%)	1 (5.3%)	
pN	pNX	12 (12.8%)	11 (14.7%)	1 (5.3%)	0.874
	pN0	17 (18.1%)	14 (18.7%)	3 (15.8%)	
	pN1	44 (46.8%)	34 (45.3%)	10 (52.6%)	
	pN2	13 (13.8%)	10 (13.3%)	3 (15.8%)	
	pN3	8 (8.5%)	6 (8.0%)	2 (10.5%)	
Adjuvant therapy	No	48 (51.1%)	39 (52.0%)	9 (47.4%)	0.887
	RT	30 (31.9%)	24 (32.0%)	6 (31.6%)	
	CCRT	16 (17.0%)	12 (16.0%)	4 (21.1%)	
Follow-up (mo)	Mean ± SD	39.4 ± 30.3	47.0 ± 29.0	9.2 ± 8.1	<0.001
	Median (IQR)	34 (13–63)	47 (20–73)	6 (3–15)	
Recurrence	No	83 (88.3%)	65 (86.7%)	18 (94.7%)	0.452
	Yes	11 (11.7%)	10 (13.3%)	1 (5.3%)	

BOT, base of tongue; CCI, Charlson Comorbidity Index; CCRT, concurrent chemoradiotherapy; CUP, carcinoma of unknown primary; ENE, extranodal extension; IQR, interquartile range; LVI, lymphovascular invasion; mRND, modified radical neck dissection; PNI, perineural invasion; PPW, posterior pharyngeal wall; RT, radiotherapy; GTS, glossotonsillar sulcus; SD, standard deviation; SND, selective neck dissection.

Overall, 94 patients were analyzed, including 69 males (73.4%) and 25 females (26.6%). The mean age was 66.5 ± 10.1 years (range, 42–95), with a median of 66 years (IQR, 59–74). The Charlson Comorbidity Index (CCI) showed a median value of 3 (IQR, 2–4).

The robotic (R; n = 75) and exoscopic (E; n = 19) groups were broadly comparable with respect to baseline demographic characteristics (**Table 1**). Patients in the R-group were slightly older than those in the E-group; however, the difference was not statistically significant (*p* = 0.051). Sex distribution and CCI did not differ significantly between the two groups (*p* = 1.000 and *p* = 0.629, respectively).

Thirteen patients had received prior treatment: 10 patients (10.6%; R = 9, E = 1) had undergone chemoradiotherapy, while 3 (3.2%; all in the R-group) had received radiotherapy alone.

The most frequent primary site was the palatine tonsil (39/94), accounting for 42.7% in the R-group and 36.8% in the E-group, followed by the base of tongue (BOT; 20/94; 21.3%; R = 16, E = 4), glossotonsillar sulcus (10/94; 10.6%; R = 6, E = 4), soft palate (7/94; 7.4%; R = 6, E = 1), and posterior pharyngeal wall (5/94; 5.3%; R = 4, E = 1). Thirteen patients (13.8%; R = 11, E = 2) were initially classified as carcinoma of unknown primary (CUP).

At diagnosis, cT classification was cT1 in 40 patients (42.6%; R = 31, E = 9), cT2 in 31 (33.0%; R = 27, E = 4), cT3 in 9 (9.6%; R = 5, E = 4), and cTis in 1 (1.1%; R = 1, E = 0). Thirteen patients (13.8%; R = 11, E = 2) were classified as cT0 due to CUP, all identified as p16-positive preoperatively. Nodal status was cN0 in 32 patients (34.0%; R = 28, E = 4), cN1 in 45 (47.9%; R = 34, E = 11), cN2 in 13 (13.8%; R = 10, E = 3), and cN3 in 4 (4.3%; R = 3, E = 1). Distant metastases (cM1) were present in 2 patients (2.1%; R = 1, E = 1).

Overall clinical stage distribution was as follows: stage 0 in 1 patient (1.1%; R-group), stage I in 59 (62.8%; R = 48, E = 11), stage II in 18 (19.1%; R = 17, E = 1), stage III in 7 (7.4%; R = 4, E = 3), and stage IV in 9 patients (9.6%; R = 5, E = 4).

Surgical procedures included 74 oropharyngectomies (78.7%; R = 60, E = 14) and 20 BOT resections (21.3%; R = 15, E = 5). Neck management consisted of selective neck dissection (SND) in 66 patients (70.2%; R = 52, E = 14) and modified radical neck dissection (mRND) in 16 (17.0%; R = 12, E = 4). The remaining 12 patients (12.8%; R = 11, E = 1) did not undergo neck surgery. Specifically, 8 patients had previously received cervical oncologic treatment (5 chemoradiotherapy, 3 radiotherapy), and 4 had undergone prior neck dissection.

Reconstruction was required in 6 patients (6.4%; R = 2, E = 4): 3 (R = 2, E = 1) received pectoralis major myocutaneous flaps, and 3 (all in the E-group) underwent radial forearm free flap reconstruction.

The mean transoral resection time was 55.8 ± 6.4 minutes in the E-group (median, 55 minutes; IQR, 49.5–61.5; range, 47–65) and 56.3 ± 9.2 minutes in the R-group (median, 58 minutes; IQR, 51.5–61.0; range, 30–81), with no statistically significant difference (*p* = 0.817).

Similarly, postoperative length of hospital stay did not differ significantly between groups, with a mean of 15.6 ± 9.7 days in the E-group (median, 12 days; IQR, 9–20.5; range, 5–36) and 13.6 ± 6.3 days in the R-group (median, 12 days; IQR, 9–15.5; range, 2–34) (*p* = 0.974).

Oral intake was resumed on the first postoperative day in all patients, initially with sterile gelled water and subsequently advanced to a regular diet as tolerated. The only exception was one patient in the E-group who had a pre-existing percutaneous endoscopic gastrostomy tube placed before surgery.

One intraoperative complication occurred (1.1%; E-group): an acute myocardial infarction, with the patient subsequently dying in the early postoperative period due to cardiac arrest, not directly related to the surgical procedure. No additional major sequelae were observed in the remaining patients.

Regarding p16 status, 64 cases (68.1%; R = 54, E = 10) were p16-positive, and 30 (31.9%; R = 21, E = 9) were p16-negative. Among p16-negative OPSCCs, histologic grading was G1 in 3 cases (R = 2, E = 1), G2 in 12 (R = 11, E = 1), and G3 in 15 (R = 8, E = 7). Perineural invasion (PNI) was present in 38 patients (40.4%; R = 32, E = 6), and lymphovascular invasion (LVI) in 44 (46.8%; R = 32, E = 12).

Pathologic T classification was pTis in 4 patients (4.3%; R = 3, E = 1), pT1 in 41 (43.6%; R = 34, E = 7), pT2 in 40 (42.6%; R = 33, E = 7), pT3 in 6 (6.4%; R = 3, E = 3), and pT4 in 3 patients (3.2%; R = 2, E = 1).

Pathologic nodal status was pNX in 12 patients (12.8%; R = 11, E = 1), pN0 in 17 (18.1%; R = 14, E = 3), pN1 in 44 (46.8%; R = 34, E = 10), pN2 in 13 (13.8%; R = 10, E = 3), and pN3 in 8 patients (8.5%; R = 6, E = 2). Among patients with pathologically assessed nodal status, extranodal extension (ENE) was documented in 26/82 patients (31.7%; R = 21/64, 32.8%; E = 5/18, 27.8%).

Surgical margins were classified as positive (presence of tumor at the inked margin), close (<1 mm), or negative (≥1 mm).

Positive margins were observed in 12 patients (12.8%): 11/75 (14.7%) in the R-group and 1/19 (5.3%) in the E-group. In the R-group, positive margins involved the deep margin in 4 cases, the superficial margin in 2, and both margins in 5. In the E-group, the single positive case involved simultaneous superficial and deep margins.

Close margins (<1 mm) were identified in 14 patients (14.9%): 9/75 (12.0%) in the R-group and 5/19 (26.3%) in the E-group.

Negative margins (≥1 mm) were recorded in 68 patients (72.3%): 55/75 (73.3%) in the R-group and 13/19 (68.4%) in the E-group. Among these, 34 cases had margins between 1 and 5 mm (R = 21, E = 13), while 34 cases had margins >5 mm, all within the R-group (**Figure 3**).

**Figure 3:**
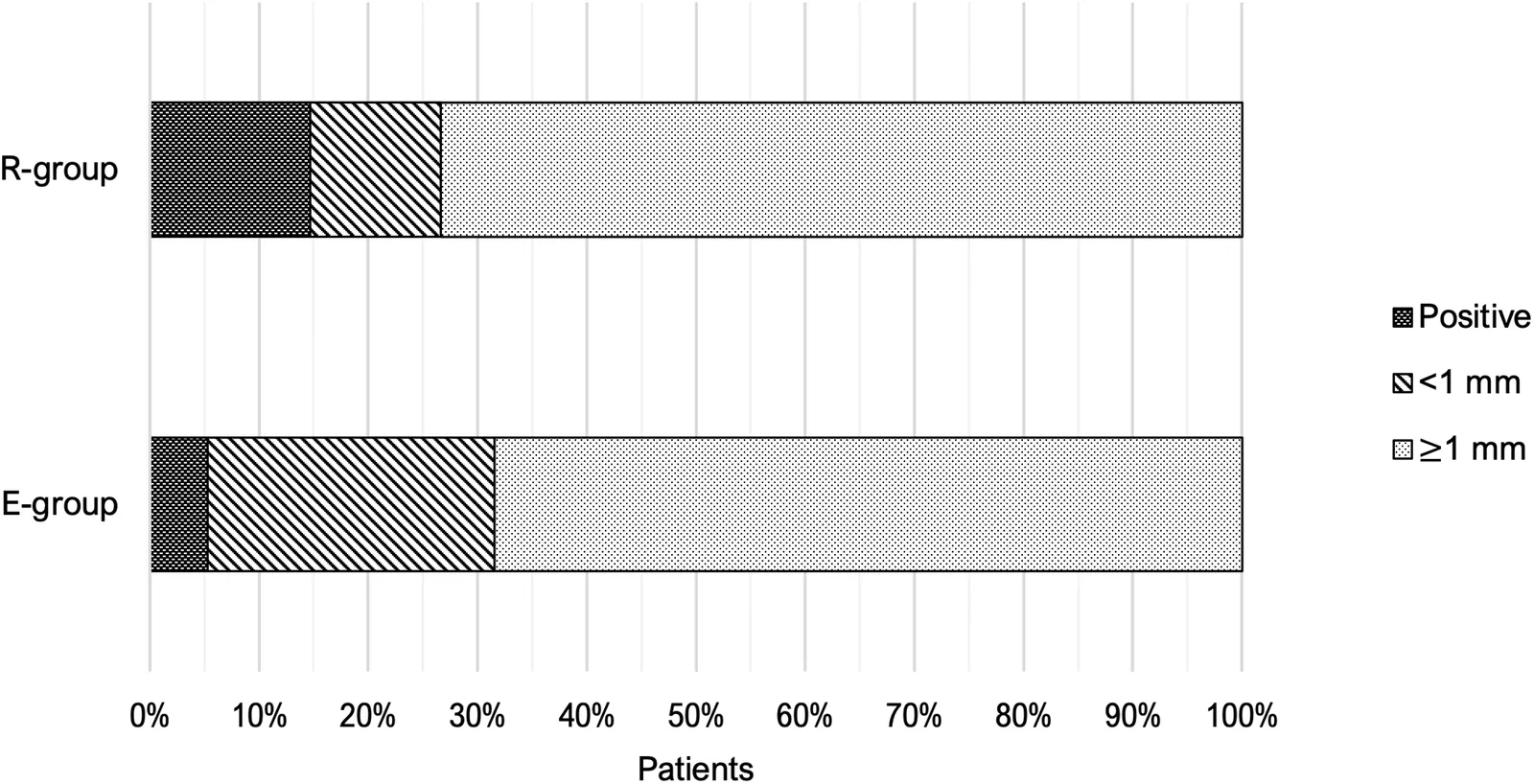
Distribution of surgical margin status in the two treatment groups.

Regarding platform-specific analysis, superficial margins were positive in 7/75 patients (9.3%) in the R-group and 1/19 (5.3%) in the E-group; <1 mm in 4/75 (5.3%) vs. 4/19 (21.1%); and ≥1 mm in 64/75 (85.3%) vs. 14/19 (73.7%). Deep margins were positive in 9/75 patients (12.0%) in the R-group vs. 1/19 (5.3%) in the E-group; <1 mm in 8/75 (10.7%) vs. 4/19 (21.1%); and ≥1 mm in 58/75 (77.3%) vs. 14/19 (73.7%).

Fisher’s exact tests showed no statistically significant differences between the two platforms either when comparing positive vs. non-positive margins (superficial: *p* = 1.000; deep: *p* = 0.681) or when comparing margins ≥1 mm vs. close/positive margins (superficial: *p* = 0.303; deep: *p* = 0.765).

To assess the potential effect of the learning curve associated with the introduction of the exoscopic platform, the first 10 consecutive procedures in each group were analyzed separately. These cases were considered to represent the early implementation phase, during which adoption of a new surgical platform might have affected technical performance and, consequently, the incidence of close or positive surgical margins. The distribution of positive, <1 mm, and ≥1 mm margins did not show statistically significant differences between groups (Fisher–Freeman–Halton exact test, *p* = 1.000). In the E-group, margins were positive in 1 case, <1 mm in 3, and ≥1 mm in 6; in the R-group, 2, 2, and 6 cases, respectively.

In the multivariable Firth penalized logistic regression analysis, advanced pathological tumor stage emerged as the only independent predictor of positive surgical margins. Compared with pTis–T2 tumors, pT3–T4 tumors were associated with approximately sixfold higher odds of positive margins (aOR 5.81, 95% CI 1.11–31.08; *p* = 0.038). In contrast, neither the surgical approach (3Des vs. TORS: aOR 0.28, 95% CI 0.02–1.57; *p* = 0.166) nor the type of transoral resection (BOT resection vs. oropharyngectomy: aOR 0.46, 95% CI 0.05–2.24; *p* = 0.366) was independently associated with an increased risk of positive margins.

Postoperatively, 30 patients (31.9%; R = 24, E = 6) received adjuvant radiotherapy, while 16 (17.0%; R = 12, E = 4) underwent concurrent chemoradiotherapy. Three patients in the R-group declined the recommended postoperative radiotherapy.

The median follow-up for the entire cohort was 34 months (IQR 13–63), with a mean of 39.4 ± 30.3 months (range, 0–107). Median follow-up was 47 months in the R-group (IQR, 20–73; mean, 47.0 ± 29.0; range, 0–107) and 6 months in the E-group (IQR, 3–15; mean, 9.2 ± 8.1; range, 0–28).

At last follow-up, 67 patients (71.3%; R = 52, E = 15) were alive with no evidence of disease (NED), 2 (2.1%; R = 2, E = 0) were alive with disease (AWD), 9 (9.6%; R = 8, E = 1) had died with disease (DWD), and 16 (17.0%; R = 13, E = 3) had died of other causes (DOC).

Overall, 11 patients (11.7%; R = 10, E = 1) developed recurrence. Among these, recurrence involved the primary site in 3 cases (R = 2, E = 1), regional nodes in 2 cases (R = 2, E = 0), and distant sites in 7 cases (R = 7, E = 0); these categories were not mutually exclusive, as one R-group patient had both regional and distant recurrence.

## 4 Discussion

In conventional pathological reporting, margins are classified as “negative (clear),” “close” or “positive” based on predefined numerical thresholds. Traditionally, a negative margin is defined as a distance of at least 5 mm between the invasive carcinoma and the inked resection edge; a close margin as 1–5 mm; and a positive margin as tumor at the ink or within less than 1 mm. These definitions are embedded within the standardized frameworks of the College of American Pathologists (CAP), the International Collaboration on Cancer Reporting (ICCR), and the World Health Organization (WHO) Classification of Tumors (2, 16–20).

In OPSCC, thresholds defining close margins range from 1 to 5 mm across authoritative sources and randomized protocols, including PATHOS, ECOG 3311, ORATOR, ORATOR2, EORTC 1420, AVOID, and SIRS (13, 21). This heterogeneity underscores the need for context-specific interpretation rather than rigid dichotomization (22).

Anatomical constraints further challenge the pursuit of uniformly wide margins, particularly along the deep plane (23). Imaging-based morphometry has shown that the posterolateral oropharyngeal wall is frequently thinner than 5 mm, especially posteriorly and in the intermuscular region, making a deep clearance of ≥5 mm anatomically unachievable in many patients (24). The distinction between clinical (*in vivo*) and pathological (*ex vivo*) margins is also substantial: post-resection tissue contraction and histopathological processing may reduce linear dimensions by 30–40%, such that a planned clinical clearance of 8–10 mm may correspond to approximately 5 mm histologically (25). However, the magnitude of this effect varies across anatomical subsites, tissue types, and measurement protocols, and no validated correction factor is currently available. Coagulation artifacts, specimen handling, and orientation may further influence margin measurements, underscoring the importance of meticulous inking, documentation, and communication between surgeons and pathologists. These sources of variability are particularly relevant given the lack of consensus regarding margin thresholds, which range from <1 mm to 5 mm across studies. Accordingly, pathological margins should be interpreted within their broader anatomical, surgical, and clinical context rather than exclusively according to fixed millimetric cutoffs (15, 26–28).

Furthermore, tissue-sampling protocols are inconsistently reported, with only isolated studies describing specimen inking, painting, or step-serial sectioning techniques. This methodological heterogeneity limits the ability of the current evidence to establish precise distance thresholds that reliably distinguish oncologically adequate from inadequate resections in transoral surgery.

Contemporary evidence indicates that the prognostic gradient across margin categories is not uniform. In TORS monotherapy series, overall rates of positive and close margins are low, with local recurrence risk rising most markedly in the presence of positive margins and a more limited separation between close and negative categories (25, 29, 30). Routine intraoperative frozen-section assessment has been associated with reduced local failure, supporting its value as a process measure for margin control (31). Salvage cohorts provide additional nuance: in previously irradiated fields, a minimum margin distance around 1 mm has emerged as a clinically relevant discriminator for local control, suggesting that sub-5 mm clearances may remain oncologically adequate in selected contexts, while highlighting the steep penalty associated with ≤1 mm deep clearance (32).

Within this framework of heterogeneous definitions, anatomical limits, and measurement variability, surgical visualization platforms may influence effective margin control. The da Vinci Xi robotic system provides wristed instrumentation and stable stereoscopic optics but entails substantial capital and per-case costs, dedicated infrastructure, and constrained availability in multi-specialty centers (33–35). Three-dimensional exoscopy (e.g., VITOM Eagle) has matured as a heads-up, team-shared alternative combining high-fidelity stereoscopic imaging with robotized optical control and compatibility with free-beam CO_2_ laser micromanipulation (36–39). CO_2_ laser resection may facilitate histopathological assessment of superficial margins by limiting electrocautery-related thermal artifacts and tissue distortion, thereby potentially improving the correspondence between clinical and pathological margin assessment. Whether such systems can achieve margin adequacy comparable to a robotic benchmark—particularly in deep planes constrained by constrictor muscle thickness—remains clinically relevant, with implications for access and scalability.

Against this background, the present study compared transoral resections performed using either a robotic platform (da Vinci Xi) or a 3D exoscope (VITOM Eagle), focusing primarily on superficial and deep pathological margin status. In the present cohort, the E-group demonstrated a positive-margin rate and a perioperative safety profile comparable to those observed with the robotic platform, with no conversions or excess major complications. As detailed in the Results, platform-specific analysis revealed no statistically significant differences in the distribution of positive, close, or negative superficial and deep margins between the two approaches. These distributions do not suggest a platform-related disadvantage in superficial or deep margin control, supporting comparable pathological radicality and surgical margin adequacy of 3D exoscopy. These findings indicate that, under the conditions evaluated in the present study, the exoscopic platform achieved pathological margin outcomes similar to those observed with the robotic approach.

These findings are consistent with the hypothesis that, within the anatomical constraints of the oropharynx and the known effects of specimen processing, effective margin control depends not only on instrument articulation but also on stable magnified visualization, coordinated assistance, and atraumatic energy delivery. The multivariable analysis further supports this interpretation. After adjustment for the type of transoral resection and pathological T category, the surgical platform was not independently associated with positive margin status, whereas pT3–T4 tumors showed approximately sixfold higher odds of margin positivity than pTis–T2 lesions. Pathological T category was therefore the only variable showing an independent statistically significant association with positive margins in the fitted model. Although the limited number of positive-margin events and the relatively small E-group preclude any formal demonstration of equivalence or non-inferiority, the lack of a detectable association between surgical platform and margin positivity, together with the observed margin distributions, supports the clinical feasibility of the exoscopic approach in appropriately selected patients. Overall, these findings suggest that margin control in the present cohort was more strongly associated with tumor extent than with the visualization platform employed.

Beyond pathological margin status, the two approaches also showed broadly similar perioperative outcomes. Transoral resection time and postoperative length of hospital stay did not differ significantly between the exoscopic and robotic groups. Likewise, early postoperative oral intake was similarly preserved in both groups. Although comprehensive functional endpoints, such as long-term swallowing function, nasogastric tube dependence, and patient-reported quality-of-life measures, were not systematically collected because of the retrospective study design, the observed early perioperative outcomes further support the clinical feasibility of 3Des.

From a practical standpoint, exoscopy also offers several advantages relevant to dissemination. The system provides high-quality shared stereoscopic visualization to the entire operating-room team, facilitating a common operative view, whereas during robotic procedures optimal stereoscopic visualization is primarily available to the console surgeon. It also allows an ergonomic heads-up posture, modular integration into existing operating rooms, and substantially lower capital and maintenance costs (9, 10, 40). Such features support broader implementation in centers without routine robotic access and may facilitate expansion of transoral oncologic programs while maintaining resection quality.

An additional strength of the present study is the adoption of a standardized specimen-handling protocol developed jointly by the surgical and pathology teams. This workflow was intended to minimize orientation errors, improve reproducibility of margin assessment, and reduce variability related to specimen processing. Given the lack of uniform tissue-sampling and reporting protocols currently described in the literature, such multidisciplinary standardization may contribute to more reliable pathological evaluation and more meaningful interpretation of surgical margins in transoral surgery (41). This methodological rigor may partially explain the consistency of margin assessment observed across both surgical platforms and should be considered when interpreting the oncological adequacy of the reported resections.

Oncologic outcomes should be interpreted with particular caution because follow-up was substantially shorter in the E-group than in the R-group. Accordingly, this study was designed primarily to assess pathological margin status and perioperative safety rather than to establish oncologic equivalence between the two surgical platforms. Additional limitations include the retrospective design, non-randomized platform allocation, marked group imbalance (R = 75 vs. E = 19), heterogeneity in prior treatments and neck management, and reliance on pathological margin measurements that remain susceptible to tissue shrinkage and specimen-processing variability. The relatively small E-group and limited number of positive-margin events also reduced the statistical power to detect potentially clinically relevant between-group differences. Because platform allocation was not randomized, selection bias related to baseline disease characteristics and clinical decision-making cannot be excluded. Although multivariable Firth logistic regression was used to adjust for the principal factors associated with margin status, residual confounding due to unmeasured or incompletely balanced variables may still have influenced the observed results.

The potential influence of the learning curve was explored by separately analyzing the first 10 consecutive cases in each group; however, this exploratory analysis should not be regarded as a formal assessment of surgical proficiency. A more rigorous characterization of the learning curve would require dedicated methods (e.g., CUSUM analysis) and a larger number of exoscopic cases. Consequently, the present data do not allow a reliable comparison of long-term local control, disease-free survival, or overall survival between the two approaches, and the generalizability of the findings should be interpreted with appropriate caution.

Prospective, adequately powered multicenter studies should incorporate standardized margin definitions, blinded pathology review, formal assessment of surgical learning curves, and long-term oncologic, functional, and patient-reported outcomes. Health-economic analyses and adjunct optical technologies (e.g., autofluorescence or NBI) may further clarify the role, scalability, and optimal application of exoscopy in contemporary transoral oncology.

## 5 Conclusion

Three-dimensional exoscopic surgery (3Des) represents a feasible minimally invasive platform for transoral resection of selected OPSCCs.

In this cohort, the VITOM Eagle system achieved pathological radicality, surgical margin adequacy, and perioperative safety comparable to those obtained with the robotic platform, while providing stable magnified visualization, shared operative field view, and ergonomic advantages that support coordinated four-handed surgery.

No statistically significant difference in early recurrence was observed; however, this finding should be interpreted with caution because of the substantially shorter follow-up in the E-group. Therefore, the present results support comparable pathological performance rather than oncologic equivalence between the two techniques.

By combining high-fidelity stereoscopic visualization, shared operative exposure, and compatibility with CO_2_ laser instrumentation, exoscopy represents a valuable complementary platform for centers without routine robotic access.

Prospective multicenter studies with larger cohorts, standardized pathological assessment, and longer follow-up are required before conclusions regarding long-term oncologic outcomes can be drawn.

## Data Availability

The original contributions presented in the study are included in the article/supplementary material. Further inquiries can be directed to the corresponding author.
